# Cognitive function in early and later life is associated with blood glucose in older individuals: analysis of the Lothian Birth Cohort of 1936

**DOI:** 10.1007/s00125-018-4645-8

**Published:** 2018-06-02

**Authors:** Drew M. Altschul, John M. Starr, Ian J. Deary

**Affiliations:** 1 Department of Psychology, The University of Edinburgh, 7 George Square, Edinburgh, EH8 9JZ UK; 2 Centre for Cognitive Ageing and Cognitive Epidemiology, The University of Edinburgh, Edinburgh, UK; 3 Geriatric Medicine Unit, Western General Hospital, Edinburgh, UK

**Keywords:** Blood glucose, Cognitive decline, Cognitive function, HbA_1c_, Older age, Type 2 diabetes

## Abstract

**Aims/hypothesis:**

The aim of this study was to examine whether cognitive function in early and later life, and decline in cognitive function from age 70 to 79 years, are associated with high blood glucose, as measured by HbA_1c_, at baseline (age 70), and changes in blood glucose from age 70 to 79.

**Methods:**

Participants (*n* = 1091) in the Lothian Birth Cohort of 1936 were examined. Fourteen tests were used to assess cognitive functions, grouped into four domains: visuospatial ability, processing speed, memory and crystallised ability. Test results, and measurements of HbA_1c_ and other health variables, were collected at each of four waves of assessment: at the mean age of 70, 73, 76 and 79 years. Data on cognitive function at age 11 was also available for this cohort. Latent growth curve modelling was performed and statistical controls for known risk factors were introduced.

**Results:**

Higher age 11 cognitive function predicted lower HbA_1c_ level at age 70 (*p* < 0.001). Higher cognitive function at age 70 was related to a comparatively smaller increase in HbA_1c_ levels from age 70 to 79 (*p* < 0.001). HbA_1c_ from age 70 to 79 did not have any consistent association with change in cognitive function from age 70 to 79. These associations survived adjustments for age, sex, education, APOE*ε4, smoking history, cardiovascular disease history, hypertension history, BMI and corrections for multiple testing.

**Conclusions/interpretation:**

Our results show that, among older individuals, high blood glucose is consistently predicted by lower cognitive function. Clinical care that examines and tracks cognitive function, while also taking the positive effects of maintaining cognitive function and emulating healthy behaviours associated with higher cognitive function into account, may be one approach for protecting at-risk individuals from elevated blood glucose and subsequent type 2 diabetes mellitus.

**Electronic supplementary material:**

The online version of this article (10.1007/s00125-018-4645-8) contains peer-reviewed but unedited supplementary material, which is available to authorised users.

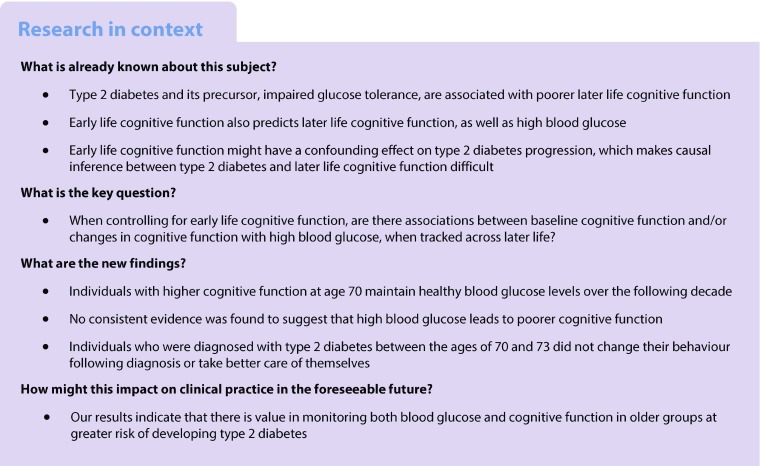



## Introduction

As the prevalence of type 2 diabetes mellitus has grown worldwide, so has the need to understand related health implications such as the link between diabetes and cognitive function. Type 2 diabetes is associated with an increased risk of cognitive decline and dysfunction, including dementia [1]. Diabetes-associated cognitive impairment is expected to become more prevalent as life expectancy for individuals with diabetes increases worldwide, highlighting a need to understand the reciprocal dynamic progression of type 2 diabetes and cognitive decline. The present study investigates the relationships between cognitive change and HbA_1c_ measurements, one of the most important risk metrics in type 2 diabetes.

Impaired glucose control, confirmed by HbA_1c_ measurement, is present in and predicts the development of type 2 diabetes, even though the syndrome may be asymptomatic and go undetected for years [2]. High HbA_1c_ is also associated with cognitive decline in older populations, in both healthy individuals [3] and those diagnosed with type 2 diabetes [4, 5]. Additionally, HbA_1c_ levels are associated with micro- and macrovascular complications [2, 6], reduction in brain volume [7, 8], and dementia [9]. The HbA_1c_–cognitive function relationship could be causative: the toxic generation of free radicals which accompanies increased HbA_1c_ may cross over into the brain, affecting cognitive functions [10].

Previous work on cognitive functioning and diabetes has focused on controlling for known risk factors such as vascular disease and the APOE*ε4 allele, as well as identifying independent risk factors such as glucose peaks [11] and insulin resistance [12] that could be linked to cognitive decline. However, whereas type 2 diabetes and high blood glucose are associated with poorer cognitive function later in life, evidence for reverse causation also exists, suggesting that lower prior cognitive function might contribute to type 2 diabetes aetiology [13]. Many samples are unable to address these types of questions because they lack a longitudinal design that tracks relevant variables, cognitive and health-related, in the same individuals across discrete waves of assessment. HbA_1c_ is a useful marker for studying the development of diabetes in this way, as it has good specificity and sensitivity to detect type 2 diabetes and also measures the severity of the disease [6, 14–16].

This study’s aim was to characterise, across the eighth decade of life, parallel changes in cognitive functions and high blood glucose (measured by HbA_1c_), and to investigate associations between baseline levels and change in these measures over time. Our study used four waves of data from the Lothian Birth Cohort of 1936 (LBC1936), a narrow age cohort of over 1000 community-dwelling people tested in four waves from age 70 to 79 years. LBC1936 also provides cognitive function data from age 11, as well as other control variables. This sample allowed us to assess two non-mutually exclusive hypotheses: higher HbA_1c_ at age 70 is associated with relatively greater cognitive decline from age 70 to 79 [17–19]; and the reverse causative hypothesis, i.e. that lower initial cognitive function and relatively greater cognitive decline are associated with subsequently higher HbA_1c_ levels [13, 20].

## Methods

### Study population

LBC1936 is a community-dwelling sample of 1091 initially healthy individuals. All were born in 1936 and were followed up in four waves of one-to-one cognitive and health testing between 2004 and 2017, at mean ages 70, 73, 76 and 79 years. Details on the background, recruitment and data collection procedures are available [21, 22]. Participants provided written informed consent. Ethics permissions were obtained from the Multicentre Research Ethics Committee for Scotland (wave 1, MREC/01/0/56), the Lothian Research Ethics Committee (wave 1, LREC/2003/2/29) and the Scotland A Research Ethics Committee (waves 2–4, 07/MRE00/58).

### Cognitive function

When they were approximately 11 years old, 1028 members of the LBC1936 sat Moray House Test No. 12, a broad cognitive ability test that included word classification, proverbs, spatial items and arithmetic. The test correlated about 0.8 with the Terman–Merrill revision of the Stanford–Binet test, providing concurrent validity [23].

In older age, cognitive function is known to decline across multiple domains [24]. Age effects also act on overall cognitive function, a superordinate factor of the lower function domains [24]. We included tests to measure three important cognitive function domains that decline with age: visuospatial ability, processing speed and memory. We also tested crystallised ability, which remains relatively stable in later life [25]. All four domains also contribute to overall cognitive ability [26]. These relationships among cognitive tests and domains are described in the Statistical Analyses section below.

Cognitive functions in waves 1–4 were assessed using 14 individually administered cognitive tests at the same clinical research facility and using the same equipment and procedure for all four waves. The tests are fully described and referenced in an open-access protocol article [21]. The visuospatial domain consisted of matrix reasoning and block design from the Wechsler Adult Intelligence Scale (WAIS) [27], and spatial span forward and backward from the Wechsler Memory Scale (WMS) [28]. Processing speed was measured through symbol search and digit symbol substitution from the WAIS, plus four-choice reaction time [29] and inspection time [30, 31]. Memory was assessed using verbal paired associates and logical memory from the WMS [28], and the letter–number sequencing and digit span backward subtests of the WAIS [27]. Crystallised ability was measured through the National Adult Reading Test [32], Wechsler Test of Adult Reading [33] and a phonemic verbal fluency test [34].

### Clinical diabetes measures

For each individual during each wave, HbA_1c_ concentrations were measured using a Menarini HA-8160 analyser (Wokingham, UK). Diabetes diagnosis status was independently recorded. Twelve individuals with type 1 diabetes were excluded from further analyses.

### Covariates

Three variable sets were evaluated as potential confounders. The first set defined our baseline control covariates: sex, age (*z* scored), age 11 cognitive function (*z* scored), and years of education (*z* scored). The second set included the available cardiovascular risk factors, obtained from interview and genetic testing: high BP history (no/yes), cardiovascular disease (CVD) history (no/yes), smoking history (current/former/never), and the presence of an APOE*ε4 allele. The third set contained only BMI (*z* scored), recorded at every testing wave and introduced to our analyses separately from the other cardiovascular variables because BMI is strongly associated with HbA_1c_ and can have a confounding effect.

### Statistical analyses

**Fig. 1:**
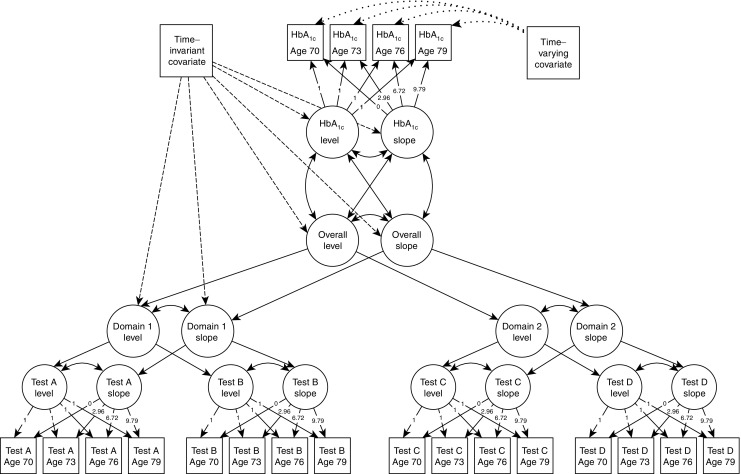
Simplified path diagram of the hierarchical factor of curves growth model of cognitive function and HbA_1c_. Circles represent latent variables and squares represent measured variables. Growth curves, including a latent level and slope factor, were estimated for each cognitive test, and the intercepts and slopes were analysed in a hierarchical model which contained domain and overall factors of both level and slope. Basis coefficients (loadings on the individual test slopes) were fixed at 0, 2.96, 6.72 and 9.79 to precisely represent the amount of time passing between assessments. All loadings on latent level were fixed at 1. Dashed lines indicate the relationships between covariates that were fixed across waves and the overall and domain latent variables. Dotted lines indicate the relationships between covariates that differed between waves and individual measurements at different waves. To preserve interpretability, not all covariate relationships are shown. Solid double-arrowed lines indicate correlations between latent variables. Solid single arrowed lines indicate the variables that load on latent variables. Although only two domains and four tests are shown here, the full model used all 14 tests in the four domains, as described in Methods

All four LBC1936 waves were analysed in latent growth curve models (LGCMs), a structural equation modelling technique that allows the user simultaneously to define and analyse multiple latent and measured variables [35]. For each variable modelled longitudinally, LGCMs model level (i.e. an intercept) and slope (i.e. a trajectory of change) variables. We modelled cognitive function using a hierarchical ‘factor of curves’ model, previously established with these data [26]. For each cognitive test we modelled a level (essentially the age 70 baseline) and a linear slope (the change between age 70 and 79, taking all four measurement occasions into account); for each cognitive domain (see Cognitive function, above) a latent level and linear slope variable were correspondingly composed of the individual tests’ latent level and latent slope variables. At the top of this cognitive hierarchy, latent levels and slopes for overall cognitive function were formed from the level and slope variables of each cognitive domain. For HbA_1c_ variables, the same approach was taken, but only a pair of level and slope variables were defined for this outcome variable, as no hierarchical structure exists. A structural diagram to illustrate these measured and latent variables is presented in Fig. 1. We also investigated quadratic slope variables for both cognitive function and HbA_1c_, but models including quadratic components either would not converge successfully, or they fit very poorly and could not be trusted to produce reliable estimates.

Put simply, HbA_1c_ level is analogous to blood glucose at the study’s beginning (age 70 years), and HbA_1c_ slope is analogous to the magnitude at which blood glucose increases over the following decade. Cognitive function level is analogous to cognitive function at age 70 and is known to be highly correlated with cognitive function at age 11 [26], demonstrating the stability of cognitive function across a lifetime. The slope of cognitive function represents the rate at which cognitive functions change (mostly decrease, in fact) over subsequent waves, which is otherwise known as cognitive decline.

Control covariates that applied to all waves of data (sex, age 11 cognitive function, years of education, APOE*ε4 status and smoking history) were regressed on to the intercept and slope variables for latent levels and slopes, including those for the domains of cognitive function, but not on to the levels or slopes of individual cognitive tests. Control covariates that were related to physical condition and could differ between waves (age, history of high BP, history of CVD, and BMI) were regressed on to the individual HbA_1c_ measurements.

Model coefficients were estimated using full information maximum likelihood, i.e. all data were used for all participants, even individuals who did not complete all waves. Standard errors were calculated using the robust Huber–White method, and *p* values were computed using the Yuan–Bentler scaled test statistic [36]. The false discovery rate correction for multiple testing was applied to the variables in each model that were not control covariates, which included the associations between the latent variables of cognitive function and its subdomains and HbA_1c_. All analyses were conducted in the R programming language, version 3.3.2 (R Foundation for Statistical Computing, Vienna, Austria), using the latent variable analysis (lavaan) package for modelling [37].

## Results

### Cohort characteristics

Demographic and clinical characteristics of the study population at all four waves are presented in Table 1. Electronic supplementary material (ESM) Table 1 presents wave 1–3 descriptions of study completers only, i.e. those individuals who remained present in wave 4.

**Table 1:** Descriptive statistics for cognitive, demographic and clinical variables

Variable	Wave 1 (*n* = 1091)		Wave 2 (*n* = 866)		Wave 3 (*n* = 697)		Wave 4 (*n* = 548)	
	T2D	No T2D	T2D	No T2D	T2D	No T2D	T2D	No T2D
Participants, *n* (%)	76 (6.97)	1015 (93.0)	84 (9.70)	782 (90.3)	75 (10.8)	622 (89.2)	66 (12.0)	482 (88.0)
Female, *n* (%)	27 (2.47)	516 (47.3)	28 (3.23)	390 (45.0)	24 (3.44)	313 (44.9)	26 (4.74)	247 (45.1)
Ever a smoker, *n* (%)	51 (4.67)	539 (49.4)	50 (5.77)	401 (46.3)	46 (6.60)	301 (43.2)	39 (7.12)	212 (38.7)
Ever had CVD, *n* (%)	32 (2.93)	236 (21.6)	35 (4.04)	215 (24.8)	34 (4.88)	202 (29.0)	25 (4.56)	179 (32.7)
Ever had high BP, *n* (%)	56 (5.13)	377 (34.6)	58 (6.70)	367 (42.4)	57 (8.18)	321 (46.1)	51 (9.31)	264 (48.2)
BMI, kg/m^2^	30.5 (4.52)	27.6 (4.28)	30.1 (5.04)	27.7 (4.32)	29.6 (4.76)	27.5 (4.39)	29.7 (5.52)	27.1 (4.34)
HbA_1c_, mmol/mol	59.0 (13.7)	40.0 (6.08)	53.1 (9.71)	37.9 (5.01)	53.2 (9.30)	39.4 (4.95)	55.1 (11.6)	38.4 (4.30)
HbA_1c_, %	7.5 (3.4)	5.8 (2.7)	7.0 (3.0)	5.6 (2.6)	7.0 (3.0)	5.8 (2.6)	7.2 (3.2)	5.7 (2.5)
Years of education	10.5 (1.09)	10.7 (1.13)	10.4 (1.02)	10.8 (1.15)	10.5 (1.06)	10.8 (1.15)	10.5 (1.09)	10.9 (1.18)
Age 11 cognitive function	94.4 (16.8)	100.0 (14.8)	96.5 (17.1)	101.0 (15)	96.7 (16.9)	102.1 (15.0)	96.5 (17.2)	103.0 (14.9)
Visuospatial ability domain								
Matrix reasoning	11.9 (5.28)	13.6 (5.10)	12.1 (4.51)	13.3 (5.00)	12.0 (5.06)	13.2 (4.88)	12.0 (4.78)	13.0 (5.06)
Block design	32.3 (11.0)	33.9 (10.3)	30.3 (9.18)	34.0 (10.1)	30.6 (8.95)	32.4 (10.1)	29.9 (8.68)	31.4 (9.75)
Spatial span	7.07 (1.65)	7.55 (1.44)	6.65 (1.70)	7.11 (1.59)	6.80 (1.74)	7.08 (1.57)	6.71 (1.48)	6.74 (1.62)
Crystallised ability domain								
NART	31.1 (9.63)	34.7 (7.98)	31.9 (8.70)	34.6 (8.08)	32.5 (8.76)	35.3 (7.89)	34.2 (9.38)	35.8 (8.01)
WTAR	37.3 (8.96)	41.3 (6.95)	38.8 (7.49)	41.2 (6.87)	39.0 (7.67)	41.3 (6.91)	40.2 (8.06)	41.8 (6.87)
Verbal fluency	40.1 (13.9)	42.6 (12.4)	39.4 (12.6)	43.6 (12.9)	40.1 (13.2)	43.2 (12.7)	41.7 (14.2)	43.9 (13.2)
Memory domain								
Logical memory	68.2 (19.0)	71.7 (17.9)	70.2 (19.6)	74.7 (17.6)	71.9 (20.6)	74.9 (19.0)	71.4 (22.6)	72.9 (20.1)
VPA	25.4 (9.08)	26.5 (9.14)	25.8 (9.52)	27.3 (9.45)	24.3 (8.87)	26.7 (9.61)	26.7 (9.96)	27.2 (9.51)
Digit span	7.30 (2.47)	7.77 (2.24)	7.27 (2.10)	7.87 (2.30)	7.19 (2.39)	7.84 (2.36)	7.08 (1.79)	7.62 (2.22)
LNS	10.2 (3.58)	11.0 (3.12)	10.3 (3.07)	11.0 (3.07)	9.7 (2.99)	10.6 (2.98)	9.4 (2.82)	10.2 (2.90)
Processing speed domain								
Symbol search	22.3 (7.45)	24.9 (6.27)	23.2 (6.49)	24.8 (6.13)	23.7 (5.89)	24.7 (6.52)	21.6 (6.37)	22.9 (6.66)
Digit–symbol coding	50.8 (12.0)	57 (12.9)	51.3 (13.2)	56.9 (12.1)	49.5 (13.6)	54.3 (12.8)	48.2 (13.5)	51.6 (12.9)
Inspection time	110 (10.4)	112 (11.0)	108 (11.5)	112 (11.8)	108 (13.0)	110 (12.5)	104 (14.3)	107 (13.5)
Reaction time	0.676 (0.107)	0.640 (0.084)	0.687 (0.122)	0.645 (0.085)	0.700 (0.116)	0.676 (0.101)	0.706 (0.112)	0.706 (0.114)
MMSE	28.1 (2.00)	28.8 (1.37)	28.4 (1.93)	28.8 (1.35)	28.1 (1.74)	28.7 (1.68)	28.2 (1.86)	28.6 (2.08)

Data are presented as *n* (%) or mean (SD)

LNS, letter–number sequencing; MMSE, Mini Mental State Examination; NART, National Adult Reading Test; T2D, type 2 diabetes, defined as self-reported physician diagnosis of diabetes; VPA, visual paired associates; WTAR, Wechsler Test of Adult Reading

Individuals with type 2 diabetes were more likely than healthy individuals to have a history of CVD or high BP (Table 1). The type 2 diabetes group included more men, as well as more current and former smokers. Individuals with type 2 diabetes had higher BMI and HbA_1c_ levels; in all waves their mean HbA_1c_ was above the diagnostic threshold of 48 mmol/mol (6.5%). Individuals with type 2 diabetes had lower age 11 cognitive function and scored more poorly than healthy individuals on all cognitive tests across all four waves.

**Fig. 2:**
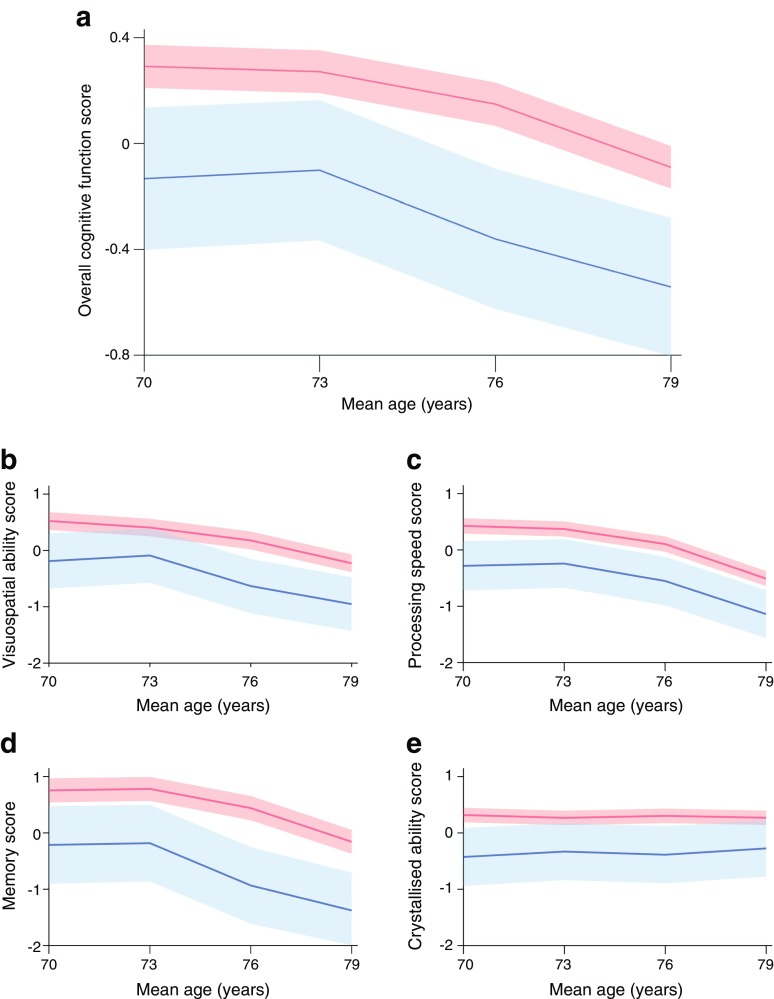
Trajectories of overall and domain-specific cognitive abilities in individuals with and without type 2 diabetes in the Lothian Birth Cohort 1936. Diabetes was defined by reported physician diagnosis or HbA_1c_ level >48 mmol/mol (6.5%) at wave 1. (**a**) Overall cognitive ability, composed of the four specific cognitive domains: visuospatial ability (**b**), processing speed (**c**), memory (**d**) and crystallised ability (**e**). Red line and 95% confidence region, individuals without diabetes; blue line and 95% confidence region, individuals with diabetes. These plots only include data from individuals with data available from all four waves of the Lothian Birth Cohort 1936 study, since including data from all individuals in these plots would bias the means towards a positive direction at older ages, making them unrepresentative of the whole sample

To illustrate cognitive decline across domains, we plotted the discrete overall cognitive function and domain scores across all four waves, with individuals categorised as either healthy or showing a clinical or biochemical (HbA_1c_ above diagnostic threshold) type 2 diabetes diagnosis at wave 1 (Fig. 2). Figure 2 represents study completers only, because including data from all study participants would bias the means of later waves in the positive direction, making plots unrepresentative of the sample. As expected, with the exception of crystallised ability, the curves show clear declines across all other cognitive domains in individuals with and without type 2 diabetes; however, cognitive function was worse in individuals with type 2 diabetes. Figure 2 is for illustration purposes only.

### Age 70–79 cognitive performance and HbA_1c_

**Fig. 3:**
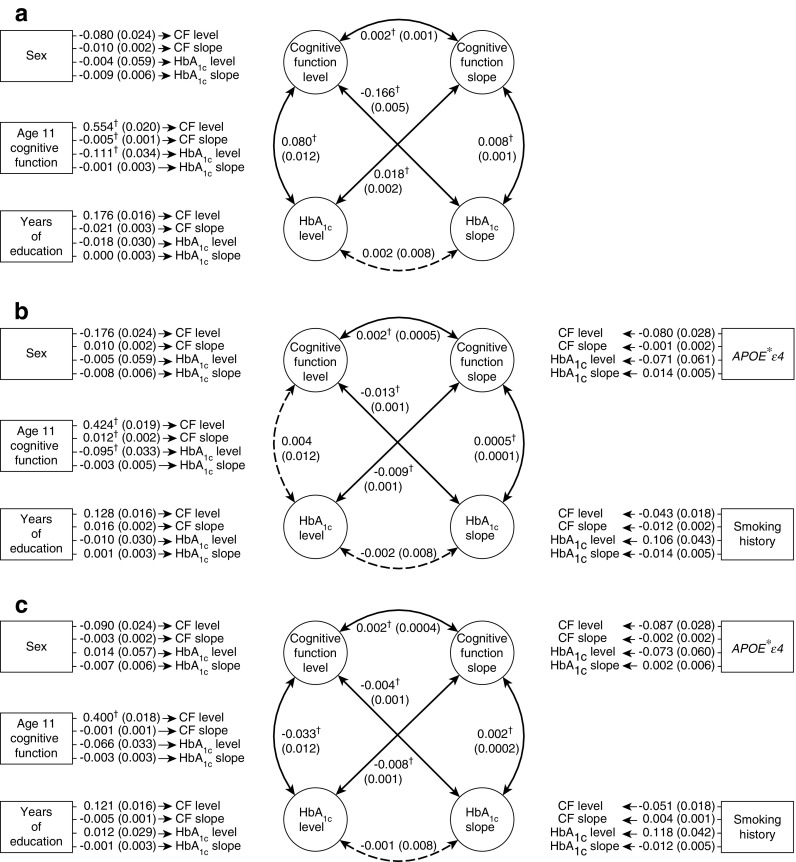
Path diagram of relationships between level and slope estimates of overall cognitive function (CF) and HbA_1c_ in the Lothian Birth Cohort 1936 from age 70 to 79 years. In (**a**), the model includes the covariates shown, as well as age. In (**b**), the model includes the covariates shown, as well as age and history of CVD and high BP, history of smoking and the presence of an APOE*ε4 allele. In (**c**), the model includes all the covariates shown, age, and history of CVD, history of high BP, smoking and APOE*ε4, as well as BMI (the latter is demonstrated as a time-varying covariate in ESM Table 2). Numbers presented are standardised estimates with SEM in parentheses. Double-headed arrows between latent variables (circles) indicate correlations. Single-headed arrows from covariates (rectangles) indicate regression effects on latent variables. Dashed lines indicate non-significant correlations, whilst solid lines and ^†^ indicate significance; The false discovery rate correction for multiple testing was applied to all tested relationships. Additional estimates for domains and controls are presented in ESM Table 2

Figure 3 shows the relationships between the level and slope estimates of HbA_1c_ and overall cognitive function. Three models are presented, with control variables added cumulatively, as described in Methods. The diagrams present the results for overall cognitive function; domain-specific results can be found in ESM Table 2. The effects of control variables on the level and slope estimates are also presented in Fig. 3, whereas disease-relevant effects of control variables, measured at each wave, are presented separately in ESM Table 2. Model fit statistics of all LGCMs indicated a good fit (root mean square error of approximation [RMSEA] <0.33; standardised root mean residual [SRMR] <0.62; comparative fit index [CFI] >0.939; Tucker–Lewis index [TLI] >0.936; see ESM Table 3 for complete fit statistics).

Age 11 cognitive function made significant contributions to both cognitive function level (*β* > 0.4, coefficient of partial determination *ρ*^2^ > 0.35) and HbA_1c_ level (*β* > −0.066, *ρ*^2^ > 0.007) at age 70; that is, higher childhood cognitive function was associated with higher cognitive function and lower HbA_1c_ concentration at age 70. More education was also associated with better cognitive function in older age, though to a lesser degree (*β* > 0.12, *ρ*^2^ > 0.026). These findings are consistent with those previously reported [13, 20, 38].

The relationship between cognitive function level at age 70 and cognitive function slope was robust and did not change when covariates were added (*r* = 0.002, *ρ*^2^ = 4 × 10^−6^), but the association was small enough that we can only infer that cognitive function level and slope have no strong relationship. This is likely due to low covariance between cognitive function level and slope; or, in other words, regardless of an individual’s starting cognitive function level, cognitive function will consistently decline over the course of the eighth decade.

Significant associations of the same sign were found between HbA_1c_ slope and both level and slope of cognitive function in models. These results indicate that individuals with higher cognitive function at age 70 maintain lower blood glucose over the following decade, and these associations remain in the presence of all control covariates. The association between cognitive function level and HbA_1c_ slope was as large as *r* = −0.166 (*ρ*^2^ = 0.03), which was found after controlling for the influence of age 11 cognitive function and education. The relationship between cognitive function slope and HbA_1c_ slope, while consistently positive and significant, was small: at most it was *r* = 0.008 (*ρ*^2^ = 6.4 × 10^−5^) in the first model (Fig. 3a), which includes the fewest controls.

Associations between HbA_1c_ level and cognitive function slope were significant but were not consistent across models and were relatively small. Moreover, the association between cognitive function level and HbA_1c_ level was not always significant, nor was it in the same direction across models. We found no significant associations between HbA_1c_ level and slope in any of these models.

Associations between HbA_1c_ growth curve estimates and cognitive function at the domain level generally followed the associations found with overall cognitive function level and slope (Fig. 3; ESM Table 2), with the exception of crystallised ability, which, even in older individuals, tends not to decline over time. In the first model, with the fewest controls, all cognitive domain variables were significantly associated with HbA_1c_ level and slope, and this held true across subsequent models, except for the slope variables of memory and crystallised ability. Decline in memory and crystallised ability was not associated with either HbA_1c_ level or slope after controls were included.

We conducted sensitivity analyses on our three structural equation models; specifically, we fit the same models, excluding individuals from a wave if they had been given any type of diabetes diagnosis. The resultant models were very similar to the models presented in Fig. 3 (ESM Table 4); effect sizes were generally reduced, and no previously small and/or unstable associations became robust in these models. We also tested whether change in cognitive function that occurred between age 11 and age 70 was associated with HbA_1c_ levels, but while age 11 cognitive function significantly predicted HbA_1c_, change in cognitive function did not (ESM Results, ESM Table 5).

### Post hoc analyses of diagnosis status interacting with cognitive function and HbA_1c_

Individuals might change their behaviour once they are aware that they have type 2 diabetes. To test whether or not higher functioning individuals may be better at caring for themselves after diagnosis, we ran a repeated-measures ANCOVA on the first two study waves, treating type 2 diabetes diagnosis, wave and overall cognitive function as independent variables and HbA_1c_ as the dependent variable. Type 2 diabetes diagnosis (*ω*^2^ = 0.338, *p* < 0.0001), cognitive function (*ω*^2^ = 0.004, *p* < 0.0001) and wave (*ω*^2^ = 0.023, *p* < 0.0001) all had individual main effects, as did the type 2 diagnosis and wave interaction (*ω*^2^ = 0.006, *p* < 0.0001). However, type 2 diagnosis and cognitive function did not significantly interact (*ω*^2^ < 0.001, *p* = 0.742), suggesting that knowledge of one’s diagnosis is not related to higher functioning individuals taking better care of themselves. No additional interactions in the model were significant either. These results were robust to the inclusion of control covariates (ESM Table 6).

In much the same way that cognitive function might behaviourally impact post-diagnosis HbA_1c_, individuals with type 2 diabetes who maintain low HbA_1c_ measurements may show cognitive differences from individuals with type 2 diabetes who do not maintain healthier HbA_1c_. We also tested this is an ANCOVA framework, making cognitive function the outcome variable, and including HbA_1c_, wave (1 or 2) and type 2 diabetes diagnosis as independent variables. We found main effects of type 2 diabetes diagnosis (*ω*^2^ = 0.014, *p* < 0.0001) and HbA_1c_ (*ω*^2^ = 0.006, *p* = 0.0001), but no effects of wave or any interactions. These effects were robust to the inclusion of covariates (ESM Table 7). Together, these findings suggest that type 2 diabetes diagnosis and HbA_1c_ are both associated with cognitive function, but neither changes in diagnosis nor whether an individual with type 2 diabetes has relatively low or high HbA_1c_ appear to be related to cognitive function.

## Discussion

The results show that in an older narrow age cohort, tested on four occasions between 70 and 79 years, lower cognitive function at 70 is associated with increases in HbA_1c_ over the following decade. Cognitive function level is negatively correlated with the slope of HbA_1c_, and this effect is robust to the inclusion of all covariates, including age 11 cognitive function. Age 11 cognitive function itself is consistently and negatively related to HbA_1c_ level, which is consistent with previous reports of this sample [20, 39]. Together, these results suggest that cognitive function consistently predicts blood glucose later in life.

Other associations between slope and level estimates of cognitive functions and HbA_1c_ were generally small and were inconsistent when different covariates were added. We found no consistent relationships between HbA_1c_ level and cognitive function slope, which represents cognitive decline. If HbA_1c_ had a causative, negative effect on cognitive function, we would expect to find this, but the largest association was 0.018 and in the opposite direction from what we would expect. The associations discussed thus far are visible in Fig. 2, where one can see that the initial level of cognitive function is lower in individuals with type 2 diabetes, though the rate of decline is not obviously different.

We found a significant association between age 11 cognitive function and HbA_1c_ level starting at age 70 and cognitive function level at age 70, but we did not find any relationships between HbA_1c_ level and age 70 cognitive function level or change in cognitive function from ages 11 and 70. This suggests that early life cognition, which is stable over the lifespan, drives the cross-sectional association between cognitive function and HbA_1c_ at age 70. In 1947, there was no HbA_1c_ measurement at age 11, so we cannot decide here whether early cognitive function is causal to worsening blood glucose across the life course to age 70, or whether cognitive functioning and blood glucose track each other through the life course.

It is notable that age 11 cognitive function was not associated with HbA_1c_ slope, but cognitive function level at age 70 was. This was surprising, given that cognitive function level at age 70 was not reliably related to HbA_1c_ level starting at the same age. However, these findings are consistent with a lead-lag effect: better cognitive functioning earlier in life predicts lower blood glucose, but only later in life.

Early cognitive function is a major life course variable that influences blood glucose and type 2 diabetes progression. In this sample, sex had few effects on level and slope outcomes. The strongest was with cognitive function level—men tended to have higher cognitive function level in this sample. Years of education display similar relationships: very small effects on all level and slope estimates other than cognitive function level, with which education showed positive relationships (*β* > 0.12).

When introduced in model B, disease variables including APOE*ε*4* and hypertension and CVD history greatly reduced the effect of cognitive function on HbA_1c_ slope. Smoking history, for example, had a notable effect on HbA_1c_ level, indicating that smokers were more likely to have higher HbA_1c_ at age 70. The impact of these variables suggests that the association between cognitive function and later measurements of HbA_1c_ is related to health behaviours. One interpretation of the results is that individuals with higher cognitive function take better care of themselves: they smoke less, are more active and have a healthier diet [40]. These associations between cognitive function and health later in life have been extensively investigated [41–43], including through diabetes epidemiology [13, 20]. However, our post hoc analyses demonstrated that the relationship between cognitive function and HbA_1c_ could not be explained by participants with higher cognitive function altering their behaviour more than those with lower cognitive function when these participants become aware of their type 2 diabetes diagnosis.

The extensive clinical and cognitive data that were available across all four waves, over a decade, are clear strengths of this study, as was the availability of a reliable cognitive function measure at an early age. Moreover, the broad range of validated cognitive tests allowed us to investigate both overall and domain level associations between cognitive function and blood glucose. As the LBC1936 is a narrow age cohort, neither cohort nor age effects could significantly bias our findings.

There were also limitations to this study that ought to be considered. First, our sample featured relatively few individuals diagnosed with type 2 diabetes, which limits the power of the statistical analyses. This issue was somewhat ameliorated by our use of HbA_1c_ as an outcome, which captures fine-grained information about diabetic and prediabetic status; nevertheless, comparatively few individuals had clinically elevated blood glucose. Second, and similarly, dropout (most likely due to mortality and frailty) was significant; almost half of the original sample did not return by the fourth wave. Whereas our models take existing and missing data into account, ultimately these missing data limit our statistical power and would particularly impact our analyses of slopes. For instance, we might not have possessed the statistical power to detect small changes in cognitive function that could have been driven by high HbA_1c_. Third, we did not have any HbA_1c_ measurements from earlier in life, so we could not control for the influence of type 2 diabetes precursors that might have existed earlier in life. However, the prevalence of type 2 diabetes in children and young adults is low [44] and would be lower still in a cohort born in 1936 [20].

HbA_1c_ not only predicts type 2 diabetes but is also associated with vascular complications [2, 6] and declines in both cognitive function [3, 4, 9] and brain volume [7, 8]. However, some prior findings are counterintuitive. For example, total brain volume was increased in a group receiving targeted glycaemic control therapy, but in spite of this no differences were found in cognitive outcomes [7]. In general, it is difficult to determine the direction of causality in the association between type 2 diabetes and cognitive decline, but the current literature suggests that having type 2 diabetes causes greater cognitive decline [11, 12, 19, 45, 46].

Our results support an alternative hypothesis, i.e. that cognitive function predicts high blood glucose; however, we stress that the two hypotheses are not mutually exclusive. The best explanation for our results and the wider body of research is that early life cognitive function contributes to impairment of glucose tolerance and onset of type 2 diabetes. Once an individual has developed diabetes, complication of the disease could lead to later impairment in cognitive function. Our results also suggest that good cognitive function continues to protect an individual from developing high blood glucose, emphasising its possible importance in ameliorating type 2 diabetes progression throughout the lifespan. Further research into the mechanisms whereby cognitive function impacts and is impacted by elevated blood glucose is warranted in the pursuit of underused strategies for identifying at-risk older individuals and protecting them from cognitive decline and type 2 diabetes.

## Electronic supplementary material

ESM (PDF 896 kb)

## Data Availability

The datasets analysed during the current study are not publicly available due to factors of individual privacy and health security, but are available from the study director of the Lothian Birth Cohorts of 1921 and 1936 on reasonable request.

## Abbreviations

CVD: Cardiovascular disease
LBC1936: Lothian Birth Cohort of 1936
LGCM: Latent growth curve model
WAIS: Wechsler Adult Intelligence Scale
WMS: Wechsler Memory Scale
