# Supplementary material for: Cognitive function in early and later life is associated with blood glucose in older individuals: analysis of the Lothian Birth Cohort of 1936
Source: Diabetologia. 2018 Jun 2;61(9):1946–55. doi: 10.1007/s00125-018-4645-8 (PMC6096629; doi:10.1007/s00125-018-4645-8)
Supplement: Supplementary file 1 — (PDF 896 kb) [file 125_2018_4645_MOESM1_ESM.pdf]

ESM Results

HbA_{1c} and cognitive function change between ages 11 and 70

Age 11 cognitive function typically explains approximately 50% of the variation in age 70 cognitive function, as assessed by the Moray House Test, which was given to all participants at age 70 to allow for direct comparisons with age 11 cognitive function. This correlation leaves room for significant cognitive change to have occurred prior to age 70. To directly test whether change in cognitive function that occurred between age 11 and age 70 was associated with HbA_{1c} at age 70, we fit a series of linear regression models. We tested if the difference between age 70 and age 11 cognitive function predicted HbA_{1c} levels. Although age 11 cognitive function significantly predicted HbA_{1c} at age 70 ($\beta < -0.06$, $\rho^2 > 0.003$, $p < 0.001$), change in cognitive function did not, regardless of which control variables were included (ESM Table 5).

ESM Table 1. Descriptive statistics for cognitive, demographic, and clinical variables for only individuals who completed all waves.

	Wave 1		Wave 2		Wave 3	
	T2D	No T2D	T2D	No T2D	T2D	No T2D
Female	9 (1.6%)	266 (48.5%)	15 (2.73%)	260 (47.4%)	21 (3.83%)	246 (44.9%)
Ever a smoker	19 (3.5%)	234 (42.7%)	22 (4.01%)	231 (42.2%)	33 (6.02%)	217 (39.6%)
Ever had CVD	11 (2.0 %)	114 (20.8%)	16 (2.92%)	139 (25.4%)	22 (4.01%)	163 (29.7%)
Ever had high BP	19 (3.5%)	176 (32.1%)	29 (5.29%)	229 (41.8%)	43 (7.85%)	243 (44.3%)
BMI	29.6 (4.55)	27.4 (4.21)	29.2 (5.13)	27.6 (4.30)	29.5 (4.98)	27.5 (4.32)
HbA_{1c} (mmol/mol)	56.9 (13.0)	39.7 (5.0)	51.9 (8.89)	37.8 (4.39)	53.8 (9.34)	39.2 (4.52)
HbA_{1c} (%)	7.4 (3.3)	5.8 (2.6)	6.9 (3.0)	5.6 (2.6)	7.1 (3.0)	5.7 (2.6)
Years of education	10.3 (1.00)	10.9 (1.18)	10.3 (0.99)	10.9 (1.18)	10.4 (1.04)	10.9 (1.18)
Age 11 cognitive function	96 (19.4)	102 (15.0)	97.8 (18.3)	102 (15.0)	97.5 (16.8)	103 (14.9)
Matrix Reasoning[a]	12.7 (5.30)	14.5 (5.00)	13.3 (4.41)	13.9 (4.94)	12.3 (4.81)	13.4 (4.91)
Block Design[a]	33.3 (9.66)	35.7 (10.2)	32.7 (8.18)	34.8 (10.2)	31 (8.45)	32.9 (9.92)
Spatial Span[a]	7.5 (1.61)	7.67 (1.41)	6.95 (1.82)	7.15 (1.56)	6.79 (1.76)	7.19 (1.55)
NART[b]	33.8 (8.91)	35.6 (7.98)	34.2 (7.88)	35.2 (8.05)	33.9 (8.68)	35.8 (7.85)
WTAR[b]	38.8 (9.40)	42 (6.82)	40.2 (6.31)	41.8 (6.71)	39.7 (7.91)	41.8 (6.78)
Verbal Fluency[b]	45.6 (15.4)	43.4 (12.6)	43.8 (13.1)	44.3 (12.9)	42.7 (13.1)	43.9 (12.7)
Logical Memory[c]	67.5 (22.8)	74.5 (16.4)	70.2 (23.4)	76.6 (16.3)	73.9 (20.8)	76 (18.2)
VPA[c]	25.4 (9.40)	28 (8.47)	27.3 (9.37)	28.4 (9.09)	26.2 (8.69)	27.4 (9.31)
Digit Span[c]	7.83 (2.97)	8.03 (2.32)	7.95 (2.14)	8.01 (2.34)	7.44 (2.55)	7.96 (2.39)
LNS[c]	11.3 (3.52)	11.4 (3.09)	11.2 (2.79)	11.4 (3.11)	10 (2.98)	10.8 (2.97)
Symbol Search[d]	23.3 (8.40)	25.8 (6.41)	24.2 (5.91)	25.6 (5.90)	24.6 (5.66)	25.3 (6.33)
Digit-Symbol Coding[d]	53.3 (12.8)	59.2 (12.3)	54.4 (13.1)	58.6 (11.8)	51.5 (13.5)	55.9 (12.0)
Inspection Time[d]	111 (7.88)	114 (10.6)	110 (9.81)	112 (11.8)	110 (11.7)	111 (11.9)
Reaction Time[d]	0.669 (0.119)	0.625 (0.075)	0.66 (0.094)	0.635 (0.081)	0.678 (0.095)	0.668 (0.095)
MMSE	28.4 (1.94)	29 (1.25)	28.6 (1.62)	28.9 (1.21)	28.2 (1.82)	28.9 (1.48)

CVD, cardiovascular disease; BP, blood pressure; NART, National Adult Reading Test; WTAR, Wechsler Test of Adult Reading; VPA, Visual Paired Associates; LNS, Letter-Number Sequencing; MMSE, Mini-Mental State Examination; T2D, individuals with type 2 diabetes; T2D was defined as self-reported physician diagnosis of diabetes. [a] part of the visuospatial ability domain, [b] part of the crystallized ability domain, [c] part of the memory domain, [d] part of the processing speed domain.

ESM Table 2. FDR corrected, standardized parameter estimates, standard errors, and p-values for LGCMs of HbA_{1c} and cognitive function.

	Model A			Model B			Model C		
Path	Estimate	SE	*p*	Estimate	SE	*p*	Estimate	SE	*p*
Tested relationships									
HbA_{1c} level ~~ HbA_{1c} slope	.002	.008	.822	-.002	.008	.849	-.001	.008	.880
HbA_{1c} level ~~ Cognitive Function level	.080	.012	< .001	.004	.012	.762	-.033	.012	.008
HbA_{1c} level ~~ Cognitive Function level (v)	-.128	.011	< .001	-.040	.011	< .001	-.009	.011	.446
HbA_{1c} level ~~ Cognitive Function level (s)	-.148	.014	< .001	-.049	.013	< .001	-.007	.012	.614
HbA_{1c} level ~~ Cognitive Function level (m)	-.053	.011	< .001	-.003	.010	.797	.019	.010	.068
HbA_{1c} level ~~ Cognitive Function level (c)	-.059	.020	.004	-.028	.019	.165	.005	.018	.826
HbA_{1c} slope ~~ Cognitive Function slope	.008	.001	< .001	.000	.000	< .001	.002	.000	< .001
HbA_{1c} slope ~~ Cognitive Function slope (v)	-.008	.001	< .001	-.001	.000	< .001	-.002	.000	< .001
HbA_{1c} slope ~~ Cognitive Function slope (s)	-.015	.001	< .001	-.001	.000	< .001	-.003	.000	< .001
HbA_{1c} slope ~~ Cognitive Function slope (m)	-.020	.001	< .001	-.002	.000	< .001	-.005	.000	< .001
HbA_{1c} slope ~~ Cognitive Function slope (c)	-.004	.001	< .001	.000	.000	< .001	-.001	.000	< .001
Cognitive Function level ~~ Cognitive Function slope	.002	.001	< .001	.002	.000	< .001	.002	.000	< .001
HbA_{1c} level ~~ Cognitive Function slope	.018	.002	< .001	-.009	.001	< .001	-.008	.001	< .001
HbA_{1c} level ~~ Cognitive Function slope (v)	-.017	.002	< .001	.010	.002	< .001	.009	.001	< .001
HbA_{1c} level ~~ Cognitive Function slope (s)	-.039	.003	< .001	.016	.002	< .001	.015	.002	< .001
HbA_{1c} level ~~ Cognitive Function slope (m)	-.047	.003	< .001	.024	.002	< .001	.022	.001	< .001
HbA_{1c} level ~~ Cognitive Function slope (c)	-.010	.002	< .001	.004	.001	< .001	.004	.001	< .001
HbA_{1c} slope ~~ Cognitive Function level	-.166	.005	< .001	-.013	.001	< .001	-.004	.001	< .001
HbA_{1c} slope ~~ Cognitive Function level (v)	.168	.005	< .001	.014	.001	< .001	.005	.001	< .001
HbA_{1c} slope ~~ Cognitive Function level (s)	.180	.008	< .001	.017	.001	< .001	.006	.001	< .001
HbA_{1c} slope ~~ Cognitive Function level (m)	.094	.007	< .001	.006	.001	< .001	.001	.001	.542
HbA_{1c} slope ~~ Cognitive Function level (c)	.066	.008	< .001	.007	.002	.001	.003	.002	.235
Cognitive Function slope ~ Age 11 Cognitive Function	-.005	.001	< .001	.012	.002	< .001	-.001	.001	.235
Cognitive Function level ~ Age 11 Cognitive Function	.554	.020	< .001	.424	.019	< .001	.400	.018	< .001
HbA_{1c} slope ~ Age 11 Cognitive Function	-.001	.003	.842	-.003	.003	.488	-.003	.003	.440
HbA_{1c} level ~ Age 11 Cognitive Function	-.111	.034	.001	-.095	.033	.005	-.066	.033	.068

Untested controls						
HbA_{1c} ~ Age (wave 1)	.082	.029	.079	.029	.080	.028
HbA_{1c} ~ Age (wave 2)	-.040	.024	-.037	.024	-.040	.023
HbA_{1c} ~ Age (wave 3)	-.086	.027	-.088	.026	-.087	.026
HbA_{1c} ~ Age (wave 4)	-.047	.034	.039	.034	.043	.033
HbA_{1c} ~ High BP (wave 1)			.154	.049	.119	.047
HbA_{1c} ~ High BP (wave 2)			.065	.036	.029	.035
HbA_{1c} ~ High BP (wave 3)			.129	.040	.094	.039
HbA_{1c} ~ High BP (wave 4)			.117	.051	.095	.049
HbA_{1c} ~ CVD (wave 1)			.110	.110	.104	.056
HbA_{1c} ~ CVD (wave 2)			.014	.014	.003	.037
HbA_{1c} ~ CVD (wave 3)			.135	.135	.124	.040
HbA_{1c} ~ CVD (wave 4)			.145	.145	.135	.052
HbA_{1c} ~ BMI (wave 1)					.244	.031
HbA_{1c} ~ BMI (wave 2)					.264	.024
HbA_{1c} ~ BMI (wave 3)					.279	.027
HbA_{1c} ~ BMI (wave 4)					.268	.032

Notation follows Lavaan syntax for path definitions: two tildes (~~) indicates a residual correlation between variables, and one tilde (~) indicates a regression path from the right-hand side to the left-hand side of the tilde. Among the tested relationships, lowercase parenthetical suffixes indicate the subdomains: v, visuospatial; s, processing speed; m, memory; c, crystallized. Untested controls present the regression effects of each variable from a given wave on the HbA_{1c} measurement at that wave. Model A includes age, sex, age 11 cognitive function, and years of education. Model B includes these covariates, plus high blood pressure (BP) history, cardiovascular disease (CVD) history, *APOE*ε4*, and smoking history. Model C includes all previously mentioned covariates and BMI (Body Mass Index).

ESM Table 3. Fit statistics of LGCMs presented in Figure 3 and Table S2.

Covariates	*χ2*	df	RMSEA	SRMR	CFI	TLI
Age, sex, age 11 cognitive function, education	4972.5	2335	.032	.061	.939	.936
+ High BP, CVD, smoking history, *APOE* ε4*	5462.0	2943	.028	.056	.942	.939
+ BMI	5739.3	3195	.027	.055	.942	.939

RMSEA = Root Mean Squared Error of Approximation, SRMR = Standardized Root Mean Square Residual, CFI = Comparative Fit Index, TLI = Tucker-Lewis Index.

ESM Table 4. FDR corrected, standardized parameter estimates, standard errors, and p-values for tested relationships in LGCMs of HbA_{1c} and cognitive function, excluding individuals with a diabetes diagnosis.

	Model A			Model B			Model C		
Path	Estimate	SE	*p*	Estimate	SE	*p*	Estimate	SE	*p*
HbA_{1c} level ~~ HbA_{1c} slope	.003	.002	0.194	-.003	.002	0.193	-.003	.002	0.171
HbA_{1c} level ~~ Cognitive Function level	-.044	.008	< .001	-.110	.008	< .001	-.066	.008	< .001
HbA_{1c} level ~~ Cognitive Function level (v)	.028	.009	0.002	.103	.009	< .001	.078	.009	< .001
HbA_{1c} level ~~ Cognitive Function level (s)	.024	.009	0.010	.108	.010	< .001	.079	.009	< .001
HbA_{1c} level ~~ Cognitive Function level (m)	.016	.007	0.020	.061	.008	< .001	.043	.007	< .001
HbA_{1c} level ~~ Cognitive Function level (c)	.011	.014	0.428	.040	.014	0.005	.023	.014	0.098
HbA_{1c} slope ~~ Cognitive Function slope	.014	.002	< .001	-.032	.004	< .001	.010	.001	< .001
HbA_{1c} slope ~~ Cognitive Function slope (v)	-.014	.002	< .001	.032	.004	< .001	-.010	.001	< .001
HbA_{1c} slope ~~ Cognitive Function slope (s)	-.029	.002	< .001	.069	.005	< .001	-.020	.002	< .001
HbA_{1c} slope ~~ Cognitive Function slope (m)	-.038	.002	< .001	.088	.005	< .001	-.026	.001	< .001
HbA_{1c} slope ~~ Cognitive Function slope (c)	-.007	.001	< .001	.017	.003	< .001	-.005	.001	< .001
Cognitive Function level ~~ Cognitive Function slope	.003	.001	< .001	.002	.000	< .001	.002	.000	< .001
HbA_{1c} level ~~ Cognitive Function slope	-.004	.001	< .001	-.027	.003	< .001	-.002	.001	0.002
HbA_{1c} level ~~ Cognitive Function slope (v)	.003	.001	< .001	.027	.003	< .001	.001	.001	0.075
HbA_{1c} level ~~ Cognitive Function slope (s)	.006	.001	< .001	.056	.004	< .001	.002	.001	0.007
HbA_{1c} level ~~ Cognitive Function slope (m)	.010	.001	< .001	.075	.004	< .001	.006	.001	< .001
HbA_{1c} level ~~ Cognitive Function slope (c)	.001	.000	0.019	.014	.002	< .001	.000	.000	0.758
HbA_{1c} slope ~~ Cognitive Function level	-.079	.002	< .001	-2.570	.070	< .001	-.056	.002	< .001
HbA_{1c} slope ~~ Cognitive Function level (v)	.076	.002	< .001	2.572	.070	< .001	.055	.002	< .001
HbA_{1c} slope ~~ Cognitive Function level (s)	.080	.004	< .001	2.717	.126	< .001	.057	.003	< .001
HbA_{1c} slope ~~ Cognitive Function level (m)	.045	.003	< .001	1.532	.103	< .001	.033	.002	< .001
HbA_{1c} slope ~~ Cognitive Function level (c)	.029	.004	< .001	1.079	.127	< .001	.022	.003	< .001
Cognitive Function slope ~ Age 11 Cognitive Function	-.047	.006	< .001	.054	.007	< .001	.006	.001	< .001
Cognitive Function level ~ Age 11 Cognitive Function	.556	.021	< .001	.379	.018	< .001	.439	.019	< .001
HbA_{1c} slope ~ Age 11 Cognitive Function	-.002	.002	0.334	.001	.002	0.816	-.001	.002	0.685
HbA_{1c} level ~ Age 11 Cognitive Function	-.057	.022	0.013	-.050	.022	0.026	.032	.022	0.154

Notation follows Lavaan syntax for path definitions: two tildes (~~) indicates a residual correlation between variables, and one tilde (~) indicates a regression path from the right-hand side to the left-hand side of the tilde. Among the tested relationships, lowercase parenthetical suffixes indicate the subdomains: v, visuospatial; s, processing speed; m, memory; c, crystallized. Untested controls present the regression effects of each variable from a given wave on the HbA_{1c} measurement at that wave. Model A includes age, sex, age 11 cognitive function, and years of education; the HbA_{1c} level latent variable has been reflected to maintain consistency with Table S2. Model B includes these covariates, plus high blood pressure (BP) history, cardiovascular disease (CVD) history, *APOE* ε4*, and smoking history. Model C includes all previously mentioned covariates and BMI (Body Mass Index); the HbA_{1c} level, slope, and cognitive function slope latent variables have been reflected to maintain consistency with Table S2.

ESM Table 5. Linear modelling predictors of age 70 HbA_{1c}

Predictor	Estimate	SE	p	Estimate	SE	p	Estimate	SE	p
Cognitive function change, age 11 to 70	-0.222	0.250	0.373	-0.221	0.248	0.374	-0.137	0.247	0.579
Age 11 cognitive function				-0.064	0.017	< 0.001	-0.055	0.017	0.001
Sex							0.540	0.502	0.282
APOE ε4* allele							-0.670	0.540	0.215
Former smoker							0.665	0.531	0.210
Current smoker							1.886	0.822	0.022
Hypertension history							2.246	0.520	< 0.001
CVD history							1.809	0.597	0.003

ESM Table 6. ANCOVAs of HbA_{1c} measurements, predicted by type 2 diabetes diagnosis, study wave, and overall cognitive function.

Predictor	Sum of squares	p	ω^2	Sum of squares	p	ω^2
T2D	640.263	< 0.001	0.338	611.006	< 0.001	0.338
CF	8.541	< 0.001	0.004	8.914	< 0.001	0.005
Wave	45.104	< 0.001	0.023	42.874	< 0.001	0.023
T2D:CF	0.074	0.742	0.000	0.006	0.927	0.000
T2D:Wave	12.443	< 0.001	0.006	11.601	< 0.001	0.006
CF:Wave	2.408	0.059	0.001	2.269	0.067	0.001
T2D:CF:Wave	1.222	0.179	0.000	0.776	0.287	0.000
Sex				0.916	0.244	0.000
CVD history				3.394	0.025	0.002
High BP history				1.003	0.223	0.000
Smoking history				7.641	0.004	0.003
APOE ε4* allele				0.276	0.522	0.000

T2D = Type 2 diabetes diagnosis, CF = Overall cognitive function, CVD = cardiovascular disease, BP = blood pressure. Data used were from waves 1 and 2, and time point was captured in the categorical wave variable.

ESM Table 7. ANCOVAs of overall cognitive function scores, predicted by type 2 diabetes diagnosis, study wave, and HbA_{1c}.

Predictor	Sum of squares	p	ω^2	Sum of squares	p	ω^2
T2D	19.98	< 0.001	0.014	18.98	< 0.001	0.013
HbA_{1c}	9.48	0.001	0.006	9.91	< 0.001	0.007
Wave	0.00	0.954	-0.001	0.00	0.971	-0.001
T2D:HbA_{1c}	1.99	0.112	0.001	1.07	0.241	0.000
T2D:Wave	0.62	0.375	0.000	0.52	0.414	0.000
HbA_{1c}:Wave	1.55	0.161	0.001	1.28	0.198	0.000
T2D:HbA_{1c}:Wave	1.54	0.162	0.001	1.63	0.147	0.001
Sex				0.02	0.878	-0.001
CVD history				12.21	< 0.001	0.008
High BP history				6.18	0.005	0.004
Smoking history				13.45	< 0.001	0.009
APOE ε4* allele				3.12	0.045	0.002

T2D = Type 2 diabetes diagnosis, HbA_{1c} = Glycated haemoglobin, CVD = cardiovascular disease, BP = blood pressure. Data used were from waves 1 and 2, and time point was captured in the categorical wave variable.
